# Unveiling the Mechanics Behind Polyimide’s Friction-Greening Phenomenon

**DOI:** 10.3390/polym16233253

**Published:** 2024-11-22

**Authors:** Zhipeng Li, Dawei Ma, Haowen Li, Baojie Zhao, Yinglong Huang, Yanbo Li

**Affiliations:** 1 Mianyang BOE Optoelectronics Technology Co., Ltd., Mianyang 621000, China; madawei@boe.com.cn (D.M.); lihaowen@boe.com.cn (H.L.); zhaobaojie@boe.com.cn (B.Z.); huangyinglong@boe.com.cn (Y.H.); 2 Institute of Fundamental and Frontier Sciences, University of Electronic Science and Technology of China, Chengdu 611000, China

**Keywords:** polyimide, friction-greening, conductivity, trap energy level

## Abstract

Polyimide (PI) has been widely used as a flexible substrate in the OLED display industry to achieve folding and other functions. However, it has unintended side effects, such as friction-greening, a green screen phenomenon caused by friction after prolonged usage. This is related to drifting TFT characteristics caused by charge accumulating in the PI in combination with the high efficiency of green pixels. In this study, the mechanism of the influence of PI structure on friction-greening was investigated. Increasing the process temperature from 350 °C to 470 °C, the chain segment structure within the PI became more regularized. Thus, the material had higher conductivity and shallower trap energy levels, which was confirmed by X-ray small angle scattering, dielectric, photoluminescence, and other methods. Under prolonged discharge conditions, less charge accumulated within PI, thus effectively mitigating the threshold voltage drift of the thin-film transistor (TFT). These results will contribute to the further optimization of the process and the development of PI materials.

## 1. Introduction

The OLED (organic light-emitting diode) is one of the mainstream modern display technologies due to its self-luminous properties [1]. Folding screens have become a focal point for capturing the high-end market as a new product introduced by major mobile phone companies. To achieve the function of folding, polymers have been chosen as the substrate to replace glass [2]. However, OLED typically use thin-film transistors (TFT) as the driver circuitry to provide the power supply to EL devices. The type of polymer used is limited by the relatively high temperatures used in the process. Polyimide (PI) is an excellent engineering plastic due to its high glass transition temperature, low coefficient of thermal expansion, and good chemical stability. Therefore, PI is widely used in the OLED display industry as a substrate of TFT [3,4,5].

However, compared to glass as a substrate, the use of PI raises some new problems. One of them is friction-greening, which is triggered by prolonged use of mobile phones. It is a key concern of consumers and mobile phone producers because the human eye is extremely sensitive to the green [6]. Many manufacturers have triggered related debates and experienced huge financial losses. As a result, many efforts have been made to address this issue. For example, electrostatic liquid [7] and shield layers [8] have been adopted to improve the performance of friction-greening. However, these methods have a side effect on the capacity or costs. More research has shown that this problem can be traced back to two main causes. One is the high efficiency of green pixels in OLED, typically 200 cd/m^2^ for green, corresponding to 150 and 10 cd/m^2^ for red and blue [9,10]. Slight current changes lead to a significant increase in the percentage of green in white light. The other is the threshold voltage (V_th_) drift of TFT [11]. A large amount of electrical charge is generated due to the constant friction between a finger and a screen, which conducts downwards and gathers in PI as a back gate. This results in a positive drift of the V_th_ with an increase in output current. Reducing the efficiency of emitters seems impractical for OLED, so it would be more cost-effective to study the association between PI and friction-greening in more depth.

In this study, three samples of PI, named sample 350, sample 450, and sample 470, were prepared by variation in the process temperature. Results from the dynamic mechanical analyzer (DMA) and static thermal mechanical analysis (TMA) indicated that the process temperature could significantly affect the free volume and the glass transition temperature (Tg), with less free volume and a higher Tg with a higher process temperature. More importantly, segments of the amorphous region shrank within the PI as the temperature increased, resulting in a more regular structure and enhanced intramolecular charge transfer (CTC), which was confirmed by X-ray small angle scattering (SAXS) and photoluminescence. Alternating current (AC) conductivity and trapping properties of PI also benefitted from these changes. As a result, the charge that accumulated in the PI could be reduced, and the V_th_ shift reduced from 1.12 V to 0.36 V when the temperature increased from 350 °C to 470 °C. The friction-greening performance also improved, and the color coordinates (CIE) changed from (0.33, 0.48) to (0.33, 0.38). This paper clarifies the relationship between PI and friction-greening for the first time. It will contribute to the process optimization of the OLED industry and the development of new kinds of PI.

## 2. Materials and Methods

Apparatus. The ultraviolet−visible (UV−vis) absorption spectrum was recorded on a TU-1901 spectrophotometer (Beijing Purkinje General Instrument Co., Ltd., Beijing, China). Photoluminescence (PL) spectrums and the PL lifetime were recorded on an FLS920 fluorescence spectrometer (Edinburgh Instruments, Livingston, UK). X-ray diffraction (XRD) patterns were recorded using an X-ray diffractometer (Bruker AXS D8 Advance, Wurzbach, Germany) with Cu Kα radiation (λ = 1.5418 Å). X-ray photoelectron spectroscopy (XPS) was performed on an ESCALAB 250 XPS using monochromatic Al Kα radiation (Thermo Fisher Scientific Co., Ltd., Waltham, MA, USA), and films were etched for a few minutes before proceeding. Fourier transform infrared spectra (FTIR) tests were conducted on a Fourier transform infrared spectrometer (Tensor II, Bruker, Wurzbach, Germany). SAXS were conducted on Xenocs Xeuss 3.0 (Xeuss, Grenoble, France). DMA and TMA were tested by the TA Q800 and the TMA Q400EM (TA Instruments, Newcastle, DE, USA). The thermodynamic analysis adopted the tensile method, and the heating rates were 3 ℃/min for DMA and 10 °C/min for TMA. The thermogravimetric analysis (TGA) was recorded on thermogravimetric analyzers coupled to a mass spectrometer (SDT 650+ Discovery MS, TA Instruments, Newcastle, DE, USA), and the heating rates were 10 °C/min. Dielectric properties were measured by Agilent4294A, and the test frequency range was 20 Hz to 20 MHz. Aluminum electrodes (1 cm in diameter) were deposited on both sides of films by vacuum evaporation. Atomic force microscopy (AFM) was carried out on a Dimension ICON (Bruker, Wurzbach, Germany).

Preparation of PI films. PI films were prepared by thermal imidization of polyamic acid (PAA). PAA was obtained by the polymerization of pyromellitic dianhydride (PMDA) and 4,4′-diaminodiphenyl ether (ODA) in N-methylpyrrolidone (NMP). Firstly, the PAA solution was coated on the glass at room temperature, and then most of the free NMP was removed by vacuum thermal evaporation. The PAA was converted to wet film via this step. Secondly, the wet film was put into the oven for polymerization via a temperature programmed method (200 °C for 1 h, 300 °C for 1 h, and 350/450/470 °C for 1 h), then cooled naturally. To avoid oxidation during thermal imidization, an N_2_ atmosphere was adopted throughout the entire process. Only the final oven temperatures were changed to study their effect on PI, and samples were marked as sample 350, sample 450, and sample 470. The structure of PI is shown in Figure S1.

## 3. Results

**Figure 1:**
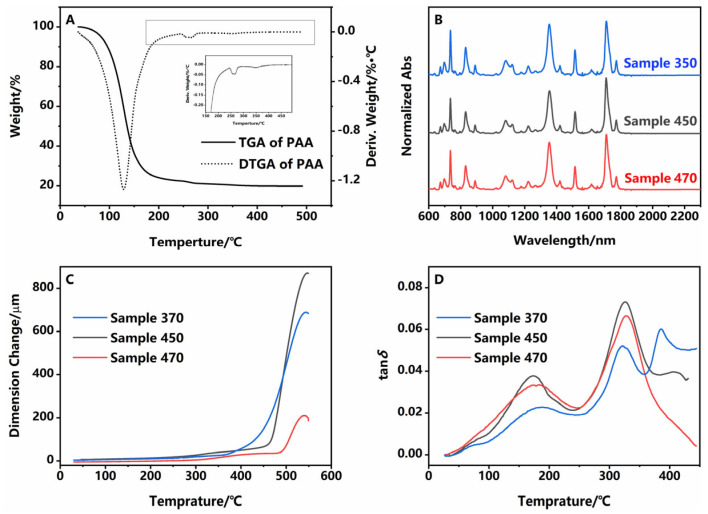
(**A**) TGA (solid line) and DTGA (dashed line) of PAA; inset: enlargement of DTGA between 150 °C and 500 °C; (**B**) FTIR of samples, (**C**) TMA and (**D**) DMA of samples. Blue lines: sample 350; gray lines: sample 450; red lines: sample 470.

The imidization of PAA mainly involves evaporating the solvent and dehydrating condensation between the carboxy and amino groups, and this process could be studied by TGA-MS methodology. As shown in Figure 1A and Figure S2, the maximum rate of weight loss was found to be at 130 °C, corresponding to the release of free NMP and the substantial evaporation of product water. The peak at 260 °C was the evaporation of bonded NMP [12,13]. A small peak at 348 °C could be found in the DTGA curve of PAA, indicating that there were still residual functional groups undergoing imidization reaction. This also confirmed that in the TGA-MS spectrum trace amounts of moisture could still be observed at this temperature. Considering the outgassing during subsequent TFT processes and the temperature limitations of the equipment, 350 °C and 470 °C were chosen as control groups, while 450 °C was currently the commonly used process temperature. To make the reaction complete, samples were kept for sufficient time, and this could be confirmed by the FTIR spectrum (Figure 1B). Additionally, 1380, 1720, and 1780 cm^−1^ should be assigned to the stretching of C-N-C, the symmetric and asymmetric stretching of C=O bonds in the imide rings. Their disappearance at 1710 and 1660 cm^−1^, which were typical absorption peaks of -COOH and -CONH- in PAA, indicated that PAA was almost converted to PI [14]. Because the C=C bond of benzene, 1510 cm^−1^, would not be affected by the imidization reaction, it could be used as the internal standard with the C-N-C peak area to calculate the degree of imidization of the three samples. Results showed that differences between samples were negligible and even lower process temperatures could ensure the reaction completely (Table 1). Therefore, the thermal stability of the material was guaranteed (Figure S3). The decomposition starting temperature (1% weightlessness) of all samples was greater than 500 °C. The elemental composition and surface roughness were also confirmed by XPS and AFM (Figures S4 and S5). Among the three samples, there was little difference in valence, content, and roughness. Thus, the chemical structure was consistent across samples.

Differences in polymer chain stacking were further explored by thermodynamics methods. TMA demonstrated that the Tg gradually increased and the coefficient of thermal expansion (CTE) gradually decreased (Figure 1C and Table 1), suggesting that as the process temperature increased from 350 °C to 470 °C, segments would be more dense, and the free volume was gradually expelled [15]. And DMA results shown in Figure 1D and Figure S6, the relaxation between 150 and 200 °C designated as β, existed in all three samples. This might be associated with molecular oscillations of benzene groups [16]. The α relaxation at higher temperatures above 300 °C, which presented the movement of segments, was quite different. For sample 350 and sample 450, there were two peaks, indicating that there were two kinds of segment structures in PI, which are more common in block polymers and semi-crystalline polymers [17,18]. However, only one peak could be found in sample 470. This suggested that one of the segments would gradually disappear with a temperature increase. And the storage modulus increased with the increase in process temperature. Based on the analysis of the above results, it could be concluded that the advanced structure of the polymers was significantly influenced by the process temperature.

**Figure 2:**
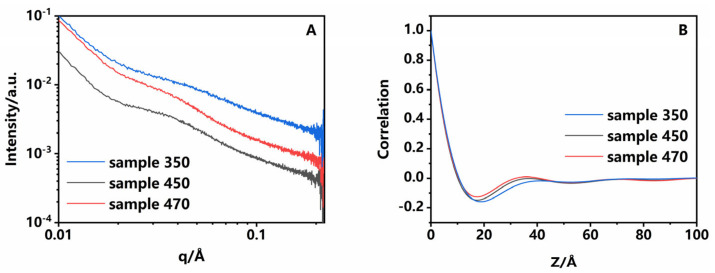
(**A**) SAXS and (**B**) correlation function of samples. Blue lines: sample 350; gray lines: sample 450; red lines: sample 470.

These segments could be further studied through SAXS and XRD. PI is a semi-crystalline polymer, and a fluctuation of electron density occurs between the crystalline and amorphous regions [19]. SAXS is an effective way to investigate this. Experimental data are presented in Figure 2A, where *q* is the scattering vector and *I(q)* is the scattering intensity. Sample 350 expressed as a broad peak with no clear peaks, indicating that poly-dispersion in a long period (crystalline + amorphous regions) was present in it. Sample 450 and sample 470 exhibited a peak that gradually shifted to the higher vector, suggesting that the periodicity became shorter as the temperature increased [20]. The correlation function can be solved by the Fourier transform of *I(q)~q* to calculate the periodicity [21], and data are presented in Figure 2B and Table 2. The long period decreased from 41 to 35.9 Å, crystalline thickness decreased from 8.16 to 7.58 Å, amorphous thickness from 32.84 to 28.32 Å, and polydispersity from 20.9 to 16.7%, whereas crystallinity increased from 19.90 to 21.11%. The crystal structure was further confirmed by XRD (Figure S7). Three samples showed two weak and broad peaks at 20 and 26°, with slightly higher crystallinity in sample 470, which is in agreement with SAXS data. This indicated that as the process temperature increased, segments of the amorphous region within the PI underwent significant shrinkage. These data indicated that as the process temperature increased, chains in the amorphous region within the PI underwent contraction and the crystalline region developed, while the crystal structure of the crystalline region remained unchanged. The overall structure became more homogeneous, which was highly consistent with thermodynamic data.

**Figure 3:**
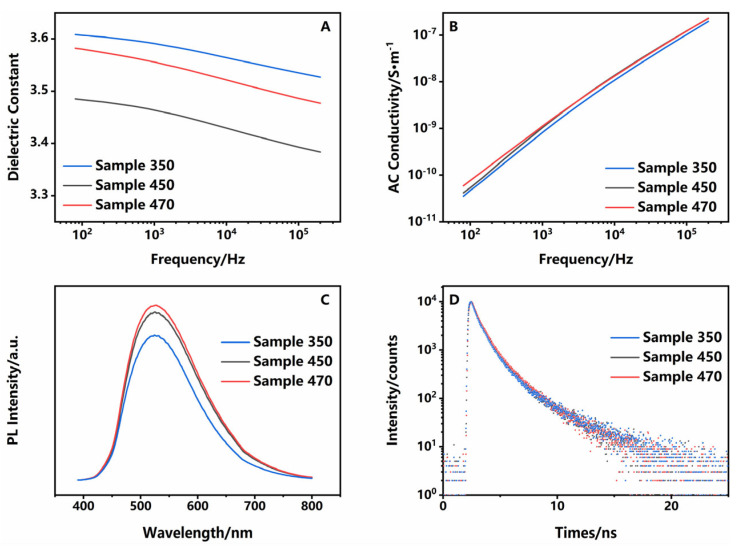
(**A**) Dielectric, (**B**) AC conductivity, (**C**) PL spectrum, and (**D**) PL lifetime decay curves of three samples. Blue lines: sample 350; gray lines: sample 450; red lines: sample 470.

Dielectric and trap properties were directly related to friction-greening. The dielectric constant was in the order of sample 350 ˃ sample 470 ˃ sample 450 (Figure 3A). Dipole polarization occurred within 20 Hz–20 MHz, which was related to the orientation and polarizability of the material. As the segments in sample 350 were relatively loosely spaced, they were more susceptible to orientation, and therefore had the highest dielectric constants. Sample 470 was also larger owing to a higher degree of crystallinity with a certain degree of orientation or higher polarizability due to CTC [22,23]. Another key parameter, conductivity, is shown in Figure 3B. A relatively high level of conductivity would allow the charge to be dispersed throughout the polymer, thus avoiding the accumulation of charge, which would have an adverse effect on TFT properties. At frequencies 100–1000 Hz, which were used in the friction-greening test, sample 470 had the relatively highest conductivity, whereas sample 350 had the lowest among the three samples. Higher conductivity could help to disperse the charge and avoid charge concentration in PI, which would have a significant effect on TFT.

CTC could also significantly affect the optical properties of PI, such as fluorescence and transmission in visible light [24,25,26]. As shown in Figure 3C, sample 470 had the strongest fluorescence intensity due to its more compact structure, with strengthened CTC. Correspondingly, the absorbance of sample 470 in the visible region was slightly reduced (Figure S8). Defects within materials were further studied by fluorescence lifetime techniques (Figure 3D and Table 3). There were two main types of traps within the PI: deep traps, caused by the chemical structure, such as the absence of the H atom in the imide ring, and shallow traps caused by the physical structure [27]. Data showed that traps in the three samples were mostly shallow, because deep traps usually lead to the quenching of fluorescence. This agreed with SAXS data. In detail, three samples could be fitted with the binary index, *I* = *A_1_*exp(*-t/τ_1_*) + *A_2_*exp(*-t/τ_2_*), where *τ_1_* denotes the intrinsic luminescence property of PI, with no significant difference between three samples, and *τ_2_* denotes the defect-related composite properties, with longer lifetimes indicating deeper trap energy levels [28,29]. Trap energy levels were deepest for sample 350 and shallowest for sample 470, and the number of defects was highest for sample 470. For shallow traps, potential barriers that the charge needed to overcome during jumping would be smaller, which reduced the density of space charge accumulation in PI [30]. For polymers with a two-phase structure, in the crystalline region the charge could easily migrate in the main chain, so it was also easier to achieve electron hopping between neighboring molecular chains due to the lower potential barriers of electrons. But it was different in the amorphous region, because of the existence of impurities and chain breakage, traps were deeper and potential barriers were higher. Therefore, it was easy to cause the build-up of the charge, resulting in the accumulation of the charge [31]. At the same time, more shallow traps can have a shielding effect on the external electric field and reduce charge injection [32]. Thus, sample 470 was expected to have the best friction-greening properties.

**Figure 4:**
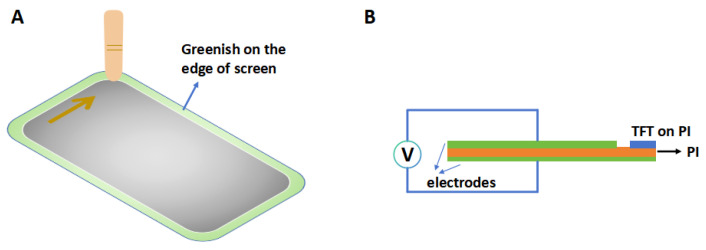
(**A**) Schematic diagram of friction-greening tests of the screen, and (**B**) test of the effect of friction-greening on TFT.

The test method for friction-greening is shown in Figure 4A. A copper rod was rubbed across the screen surface in a defined path for several hours, or an electric field could be used to generate a direct charge to reduce the time required for the test. CIE and brightness were measured in a specific image after the tests. As for TFT tests, a TEG was fabricated on the PI, and the charge was injected by AC signals (180 Hz, 300 V) via two electrodes (Figure 4B). Next, changes in TFT characteristics were tested. The charge generated during the test passed through the film layer at the screen edge and eventually accumulated in the PI, which would trigger the bottom gate-like effects model to affect the characteristics of the TFT [11]. Typically, green had the highest light-emitting efficiency, so it was usually displayed with a greenish color. TFT characteristics and CIEs of different samples after testing are shown in Table 4. The ΔV_th_ of sample 470 was as low as 0.36 V, but for sample 350 it could be as high as 1.12 V. In contrast, when glass was chosen as the substrate, the voltage was around 0.02 V. Compared to the standard white spot (0.33, 0.33), sample 350 showed a distinct green color. However, sample 470 showed only a slight green.

## 4. Conclusions

In summary, samples 350, 450, and 470 were prepared by varying the final process temperature. Differences in structure were explored and the mechanism of their effect on friction-greening was analyzed. Results indicate that process temperature could significantly affect the arrangement of segments, but had little effect on chemical structure, chemical bonding, elemental composition, and surface roughness. The long-period structure of the PI became shorter and more homogeneous as the process temperature increased. The main manifestation was the contraction of the amorphous zone, with a slight increase in crystallinity. As a result, CTC was enhanced, leading to higher conductivity and fluorescence intensity. On the other hand, the shallower trap occurred in the material. Therefore, it could reduce the accumulation of internal charges in the material. The V_th_ drift reduced from 1.12 V to 0.36 V, and the CIE improved from (0.33, 0.48) to (0.33, 0.38) with less intensity change. This result paves the way for the further development of materials.

## Figures and Tables

**Table 1:** Degree of imidization and Tg of three samples.

	C−N−C	Tg_1_/°C	Tg_2_/°C	CTE/ppm·°C^−1^
sample 350	5.02	322.81/387.52	443.11	15.64
sample 450	4.96	327.05/408.34	468.33	6.464
sample 470	5.05	328.06	491.17	4.297

Tg_1_ was from DMA, Tg_2_ was from TMA, and range of CTE was 50−450 °C.

**Table 2:** Correlation function data of three samples.

	Long Periodrazmak/Å	Crystalline Thicknessrazmak/Å	Crystallinityrazmak/%	Polydispersityrazmak/%
sample 350	41	8.16	19.90	20.9
sample 450	36.9	7.63	20.68	19.8
sample 470	35.9	7.58	21.11	16.7

**Table 3:** Correlation function data of three samples.

	τ_1_/ns	A_1_/Counts	τ_2_/ns	A_2_/Counts
sample 350	0.6446	13,361	2.9807	1149.5
sample 450	0.6111	13,290	2.4114	1786.6
sample 470	0.6231	12,771	2.3449	1908.1

**Table 4:** ΔV_th_ and CIE of different samples after tests.

	ΔV_th_/V	CIE	Intensity/Nit
sample 350	1.12	0.33, 0.48	0.40
sample 450	0.78	0.32, 0.41	0.23
sample 470	0.36	0.33, 0.38	0.15

## Data Availability

The original contributions presented in the study are included in the article/Supplementary Material, further inquiries can be directed to the corresponding author.

## Supplementary Materials

The following supporting information can be downloaded at: https://www.mdpi.com/article/10.3390/polym16233253/s1, Figure S1: Chemical structure of PI; Figure S2: TGA-MS of PAA; Figure S3: TGA curves of three samples; Figure S4: XPS of three samples; Figure S5: AFM images of three samples; Figure S6: Storage modulus curves of three PI samples; Figure S7: XRD of three samples; Figure S8: UV-visible spectrum of three samples.
