# Supplementary material for: Unveiling the Mechanics Behind Polyimide’s Friction-Greening Phenomenon
Source: Polymers (Basel). 2024 Nov 22;16(23):3253. doi: 10.3390/polym16233253 (PMC11644810; doi:10.3390/polym16233253)
Supplement: Supplementary file 1 [file polymers-16-03253-s001.zip › polymers-3329561-supplementary.pdf]

Supplementary Materials

Unveiling the Mechanics Behind Polyimide's Friction-Greening Phenomenon

Zhipeng Li [1,2,*], Dawei Ma [1], Haowen Li [1], Baojie Zhao [1], Yinglong Huang [1] and Yanbo Li [2,*]

[1] Mianyang BOE Optoelectronics Technology Co., Ltd., Mianyang 621000, China
[2] Institute of Fundamental and Frontier Sciences, University of Electronic Science and Technology of China, Chengdu 611000, China
* Correspondence: lizhipeng-b11@boe.com.cn (Z.L.); yanboli@uestc.edu.cn (Y.L.)

Contents

1. Chemical structure of PI....2
2. TGA-MS of PAA....2
3. TGA curves of three samples....2
4. XPS of three samples....3
5. AFM images of three samples. 3
6. Storage modulus curves of three PI samples 3
7. XRD of three samples 4
8. UV-visible spectrum of three samples 4

1. Chemical structure of PI

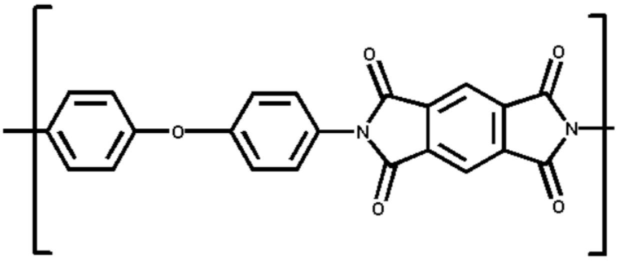

Figure S1. Chemical structure of PI.

2. TGA-MS of PAA

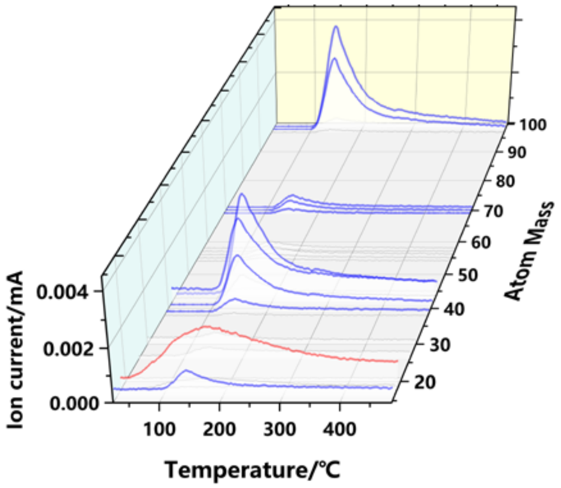

Figure S2. TGA-MS of PAA. Blue lines: NMP; red line:s H_2O.

3. TGA curves of three samples

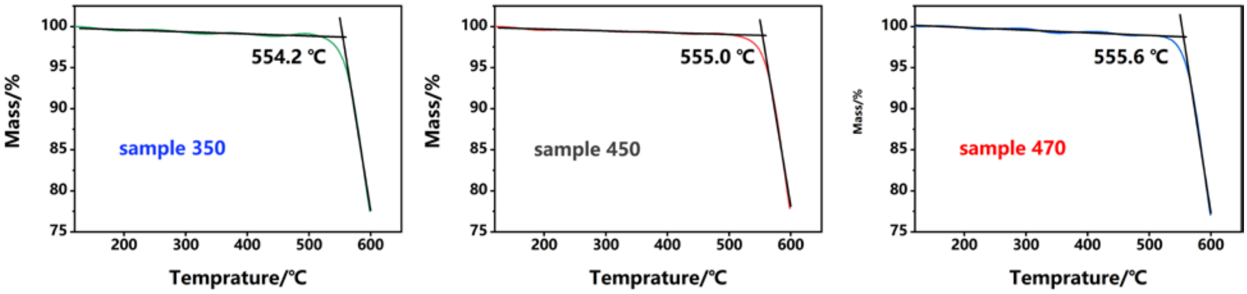

Figure S3. TGA curves of three samples.

4. XPS of three samples.

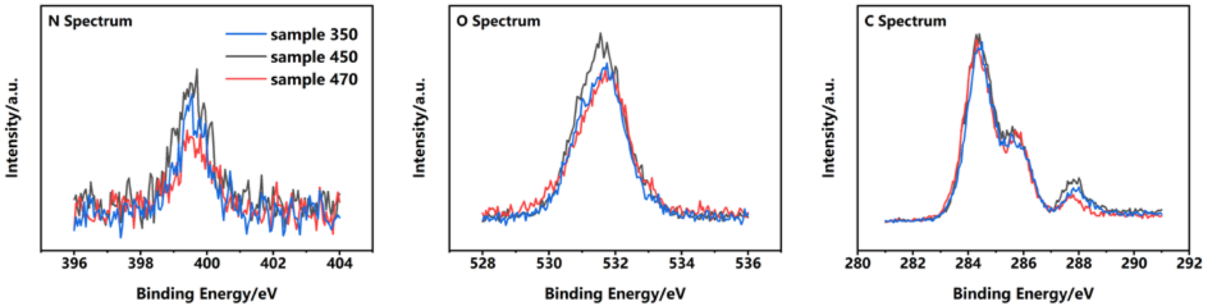

Figure S4. XPS of three samples. Blue lines: sample 350; gray lines: sample 450; red lines: sample 470.

5. AFM images of three samples.

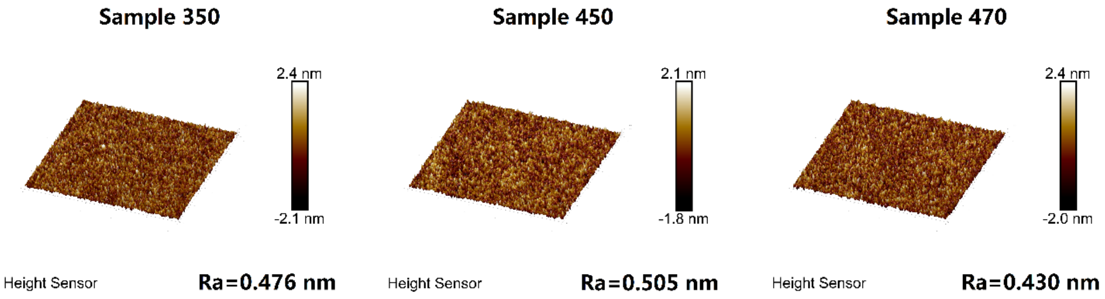

Figure S5. AFM images of three samples.

6. Storage modulus curves of three PI samples

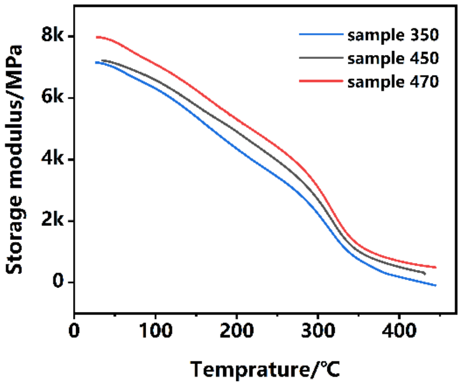

Figure S6. Storage modulus curves of three PI samples.

7. XRD of three samples

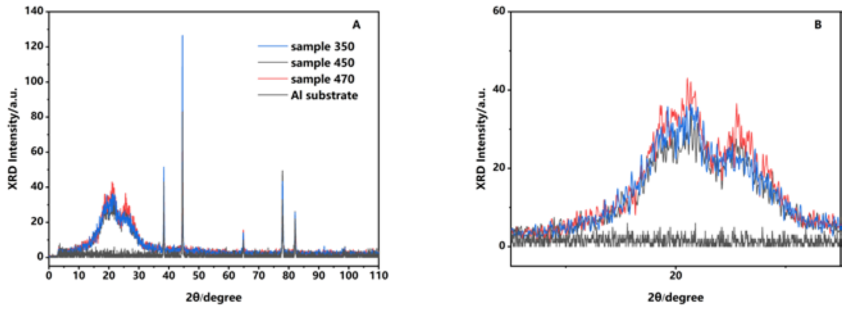

Figure S7. XRD of three samples. Blue lines: sample 350; gray lines: sample 450; red lines: sample 470; black lines: Al substrate.

8. UV-visible spectrum of three samples

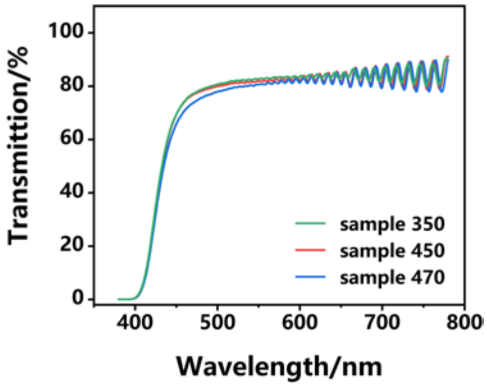

Figure S8. UV-visible spectrum of three samples. Blue lines: sample 350; gray lines: sample 450; red lines: sample 470; black lines: Al substrate.
